# Synthesis of a Fluorescent Solvatochromic Resin Using Suzuki–Miyaura Cross-Coupling and Its Optical Waveguide Spectra to Measure the Solvent Polarity on the Surface

**DOI:** 10.3390/ma13204483

**Published:** 2020-10-10

**Authors:** Yu Otsuka, Guanglei Li, Hiromi Takahashi, Hisashi Satoh, Koji Yamada

**Affiliations:** 1 Division of Materials Science, Graduate School of Environmental Science, Hokkaido University, Sapporo, Hokkaido 060-0810, Japan; y_outsuka214@eis.hokudai.ac.jp; 2 School of Food Science and Technology, Nanjing University of Finance & Economic, Nanjing 210023, China; ligliht@gmail.com; 3 ATR Scientists Partners Inc., 3-27-13, Maehara, Koganei, Tokyo 184-0013, Japan; atr.scientists.partners.inc.1995@gmail.com; 4 Division of Environmental Engineering, Faculty of Engineering, Hokkaido University, Sapporo, Hokkaido 060-8628, Japan; qsatoh@eng.hokudai.ac.jp; 5 Division of Materials Science, Faculty of Environmental Earth Science, Hokkaido University, Sapporo, Hokkaido 060-0810, Japan

**Keywords:** Suzuki–Miyaura cross-coupling, solid-phase synthesis, optical waveguide spectrometry

## Abstract

We have established a novel analytical method for solvent polarity on resin surface by combining the synthesis of fluorescent solvatochromic resin with optical waveguide spectrometry. The fluorescent solvatochromic resin was obtained via Suzuki–Miyaura cross-coupling between 4-iodobenzoic acid immobilized on Wang resin and 5-[4-(*N,N*-dihexylamino)phenyl]-2-thienylboronic acid *N*-methyl-iminodiacetic acid (MIDA) ester. The optical waveguide spectrometry studies on the resin showed a strong fluorescent solvatochromism in various organic solvents.

## 1. Introduction

The solid-phase synthesis technique was originally developed in 1959 by R. B. Merrifield to synthesize a tetrapeptide [1]. The major advantage of this method is the easy purification of the desired compounds on an insoluble resin by filtration and washing [2]. Generally, the solid-phase synthesis of bioactive molecules, such as oligonucleotides, peptides, and oligosaccharides, involves the use of highly reactive activators, such as phosphoramidite and carbodiimides, to form non-conjugated bonds [3,4,5]. The Suzuki–Miyaura cross-coupling is also a candidate for the elementary reaction in the solid-phase synthesis due to its high reactivity [6,7,8]. This coupling reaction has been applied for combinatorial chemistry to develop π-conjugated compounds by solid-phase synthesis [9,10,11].

In general, non-conjugated compounds prepared through solid-phase synthesis were used to be fully identified using several analytical techniques such as nuclear magnetic resonance, high-performance liquid chromatography, and mass spectrometry after cleavage from the solid-phase [12]. For π-conjugated compounds, their specific photophysical properties such as fluorescence and absorption characteristics could be used to identify the resin modification sensitively without cleavage [13,14]. Herein, we report the synthesis of a fluorophore modified resin and its uses for a highly sensitive analytical method to identification of the synthesized compound based on its photophysical properties. Our previously reported fluorescent solvatochromic dyes exhibit strong absorption and emission bands in the visible light region, and the fluorescence color changes depending on the solvent polarity [15,16,17]. Thus, photophysical properties of fluorescent solvatochromic dye are suitable as an indicator to identify a synthesized compound on resin surface. Wang resin has been frequently used as a solid-phase for the synthesis of small π-conjugated compounds [18,19,20]. Furthermore, along with these reasons, owing to a good swelling ability in various organic solvents, a simple cleavage reaction proceeding in acidic or basic conditions, the ability to purify and identify the cleaved product using the usual method, and the use of synthesized dye as a reference compound, we chose the Wang resin as a solid phase [21]. To measure the photophysical properties of the synthesized resin, we used an optical waveguide spectrometer with a xenon light source. Optical waveguide spectroscopy is known to be a powerful tool for the detection of optical materials because of its good selectivity, high sensitivity, and simultaneous detection of absorption and emission spectra [22]. It has also been applied to study optical materials and development of an optical sensors [23,24,25,26,27]. In this work, we established an optical measurement system including the synthesis of a fluorescent solvatochromic resin using the Suzuki–Miyaura cross-coupling reaction and the analysis of its specific photophysical properties as the identification of the synthesized compound on the resin surface.

## 2. Materials and Methods

### 2.1. General

All chemicals were reagent grade and used without further purification. Reagents were purchased from Wako Pure Chemical Industries (Osaka, Japan) with the following exceptions: Wang resin was purchased from Watanabe Chemical Industries, Ltd. (Hiroshima, Japan), *N*-methyliminodiacetic acid and bis(pinacolato)diboron were purchased from Tokyo Chemical Industry Co., Ltd., (Tokyo, Japan) 2-dicyclohexylphosphino-2’,6’-dimethoxybiphenyl (SPhos) and *N,N*-dimethyl-4-aminopyridine (DMAP) were purchased from Sigma-Aldrich Japan Co., LLC (Tokyo, Japan), and 5-bromothiophene-2-boronic acid was purchased from Combi–Blocks Inc. (San Diego, CA, USA). The reactions were monitored by thin-layer chromatography (TLC) on a pre-coated plate of silica gel 60F_254_ (layer thickness, 0.25 mm; E. Merck, Darmstadt, Germany). TLC plates were visualized with UV. Wakosil^®^ C-300 (0.04–0.06 mm, Wako Pure Chemical Industries) was used for open column chromatography.

We recoded the nuclear magnetic resonance (NMR) spectra on a JEOL instrument (Tokyo, Japan) at 400 MHz for ^1^H NMR in CDCl_3_ and acetone-d_6_ with tetramethylsilane (TMS) as internal reference. High-resolution electrospray ionization (ESI) mass spectra were recorded on a Thermo Scientific Exactive spectrometer (Thermo Fisher Scientific, Waltham, MA, USA). The attenuated total reflection infrared (ATR-IR) spectra of the synthesized beads and compounds were obtained using a Nicolet iS10 FT-IR spectrometer (Thermo Fisher Scientific).

### 2.2. Measurements

All spectroscopic measurements were performed in spectrometric-grade solvents from Wako Pure Chemical Industries.

**Figure 1:**
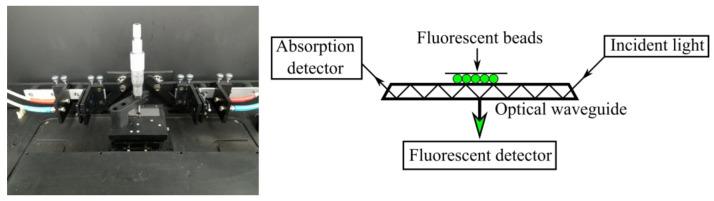
A Schematic view of the optical waveguide spectrometer with micrometer (**left**) and measurement process of optical waveguide spectrometry (**right**).

For the solid sample, the absorption and emission spectra were obtained using an optical waveguide spectrometer (PLT-5000SLC, System-Instruments Co., Tokyo, Japan) with a xenon 150 W light source. A micrometer was used to prevent the float and spread of a synthesized resin on the optical waveguide (a schematic view of the optical waveguide spectrometer is shown in Figure 1). A glass plate (65 × 20 mm with a thickness of 400 μm and a refractive index of 1.81) was used as the optical waveguide. The incident angle (normal to the waveguide surface) was fixed at 56.5° and the angle of detection (normal to the waveguide surface) was fixed at 46.2° when toluene was used as the solvent. In other solvents, the incident angle was fixed at 53.6° and the angle of detection was fixed at 44.0°. For the solution sample, the absorption spectra were recorded on a JASCO V–560 spectrometer and the fluorescence spectroscopic studies were performed on a Hitachi F-4500. The samples for the absorption and emission measurements were contained in 1 × 1 cm quartz cell. The concentration of the dye 7 solution was adjusted to 50 μmol L^−1^ for measuring the absorption spectra and to 5 μmol L^−1^ for the emission spectra. The slit width was 5 nm for both excitation and emission. All the fluorescence images were obtained on a Nikon eclipse 50i fluorescence microscope with a DS–Fi2–L3 microscope camera (Nikon) and a 395 nm UV hand lamp as the excitation light source. The fluorescence quantum yields of the samples were calculated using a Quantaurus-QY (Hamamatsu Photonics, Hamamatsu, Japan).

### 2.3. Synthetic Procedure

#### 2.3.1. Loading of 4-iodobenzoic Acid to Wang Resin (1)

Wang resin (0.500 g, 0.790 mmol g^−1^, 0.395 mmol) was suspended in anhydrous dichloromethane (7.50 mL) in a 15 mL of screw vial. 4-iodobenzoic acid (0.294 g, 1.19 mmol) and *N,N*-dicyclohexylcarbodiimide (0.245 g, 1.19 mmol) were added to the resin mixture. Then, DMAP (5.00 mg, 0.0409 mmol) in a minimum amount of dichloromethane solution was added dropwise at that mixture. The mixture was shaken for 24 h at room temperature. The resin was filtered and washed with dichloromethane (DCM), tetrahydrofuran (THF), *N*,*N*-dimethylformamide (DMF) and MeOH (×3) and finally dried under vacuum. The loading of this resin was determined to be 0.545 mmol g^−1^ by cleaving with 10% TFA/DCM solution.

#### 2.3.2. Synthesis of N,N-dihexyl-4-iodoaniline (2)

4-iodoaniline (2.00 g, 9.13 mmol), 1-iodohexane (6.39 g, 30.1 mmol) and Na_2_CO_3_ (1.74 g, 16.4 mmol) in DMF 25.0 mL, and flushed with nitrogen gas in a 50 mL of round-flask. The mixture was heated at 95 °C for 20 h. After cooling to r.t., the mixture was poured into H_2_O 100 mL and extracted with ethyl acetate 50.0 mL for three times. The combined organic layers were washed with H_2_O 50.0 mL for two times, and dried over Na_2_SO_4_. The concentrated residue was purified by silica column chromatography (hexane) to give compound **2** as a colorless oil (2.56 g, 72.4%). ^1^H NMR (400 MHz, CDCl_3_) δ (ppm) 7.39 (d, 2H, *J* = 8.1 Hz), 6.40 (d, 2H, *J* = 8.1 Hz), 3.20 (t, 4H, *J* = 7.5 Hz), 1.52 (m, 4H), 1.30 (m, 12H), 0.89 (t, 6H, *J* = 6.8 Hz); ESI–HRMS (m/z) [M+H]^+^ calcd for C_18_H_31_NI 388.14957, found: 388.14943.

#### 2.3.3. Synthesis of N,N-dihexyl-4-(4,4,5,5-tetramethyl-1,3,2-dioxaborolan-2-yl)aniline (3)

Compound 2 (1.00 g, 2.58 mmol), KOAc (1.27 g, 12.9 mmol), bis(pinacolato)diboron (1.31 g, 5.16 mmol) and PdCl_2_(dppf)CH_2_Cl_2_ (0.105 g, 0.129 mmol) were dissolved in DMF 11.0 mL, and flushed with nitrogen gas in a 30 mL of round-flask. Then, the mixture was stirred at 90 °C for 21 h. After cooling to r.t., the mixture was neutralized by 1N aq. HCl solution and extracted with 20.0 mL of ethyl acetate. The organic layers were washed with sat. aq. NaCl solution and sat. aq. NaHCO_3_ solution, and dried over Na_2_SO_4_. The residue was purified by silica column chromatography (1% ethyl acetate in hexane) to give compound **3** as a colorless oil (0.461 g, 46.1%). ^1^H NMR (400 MHz, CDCl_3_) δ (ppm) 7.64 (d, 2H, *J* = 8.8 Hz), 6.59 (d, 2H, *J* = 8.8 Hz), 3.27 (t, 4H, *J* = 7.8 Hz), 1.56 (m, 4H), 1.31 (m, 12H), 0.89 (t, 6H, *J* = 6.8 Hz); ESI–HRMS (m/z) [M+H]^+^ calcd for C_24_H_43_O_2_N^10^B: 387.34177, found: 387.34206.

#### 2.3.4. Synthesis of 5-bromo-2-thienylboronic Acid N-methyliminodiacetic Acid Ester (4)

5-bromothiophene-2-boronic acid (1.40 g, 6.77 mmol), *N*-methyliminodiacetic acid (*N*-MIDA, 1.05 g, 7.14 mmol) were dissolved in toluene 50 mL and DMSO 5 mL mixture in a 100 mL of round-flask, and flushed with nitrogen gas. The flask was fitted with a Dean–Stark trap, and stirring at 95 °C for 24 h. The mixture was concentrated under reduced pressure, and then the residue was poured into H_2_O to form precipitate. The resulting powder was filtered and washed with H_2_O and diethyl ester at several times to give compound **4** as a white sold (1.76 g, 80.9%). ^1^H NMR (400 MHz, acetone-d_6_) δ (ppm) 7.19 (d, 1H, *J* = 3.5 Hz), 7.13 (d, 1H, *J* = 3.5 Hz), 4.39 (d, 2H, *J* = 17 Hz), 4.20 (d, 2H, *J* = 17 Hz), 2.92 (s, 3H); ESI–HRMS (m/z) [M+Na]^+^ calcd for C_9_H_9_O_4_N^10^BBrNaS: 338.94572, found: 338.94590.

#### 2.3.5. Synthesis of 5-[4-(N,N-dihexylamino)phenyl]-2-thienylboronic Acid MIDA Ester (5)

Compound **3** (71.5 mg, 0.185 mmol), compound **4** (40.0 mg, 0.126 mmol), K_3_PO_4_ (80.0 mg, 0.377 mmol) and PdCl_2_(dppf)CH_2_Cl_2_ (4.00 mg, 4.90 μmol) in THF 2 mL and H_2_O 0.0110 mL were dissolved to a 10 mL of round-flask, and flushed with nitrogen gas. The mixture was stirring at 20 °C for 24 h. The mixture was poured into H_2_O 10.0 mL and extracted with ethyl acetate 20.0 mL for three times. The combined organic layers were washed with H_2_O 10.0 mL for two times, and dried over Na_2_SO_4_. The concentrated residue was purified by silica column chromatography (45% acetone in hexane) to give compound **5** as a yellow oil (29.3 mg, 46.7%). ^1^H NMR (400 MHz, acetone-d_6_) δ (ppm) 7.48 (d, 2H, *J* = 8.8 Hz), 7.21 (d, 1H, *J* = 3.5 Hz), 7.14 (d, 1H, *J* = 3.5 Hz), 6.70(d, 2H, *J* = 8.8 Hz), 4.35 (d, 2H, *J* = 17 Hz), 4.15 (d, 2H, *J* = 17 Hz), 3.35 (t, 4H, *J* = 7.6 Hz), 2.86 (s, 3H), 1.68-1.53 (m, 4H), 1.34 (m, 12H), 0.89 (t, 6H, *J* = 7.0 Hz); ESI–HRMS (m/z) [M+H]^+^ calcd for C_27_H_40_O_4_N_2_^10^BS: 498.28327, found: 498.28364.

#### 2.3.6. Synthesis of Fluorescent Solvatochromic Dye Substituted Resin (6)

0.150 g of resin **1** (0.545 mmol g^−1^, 81.8 μmol) was suspended in dioxane 3.00 mL in a 15 mL of round-flask. Compound **5** (59.0 mg, 0.118 μmol), SPhos (2.00 mg, 4.87 μmol) and Pd(OAc)_2_ (0.600 mg, 2.67 μmol) were added to the flask, and flushed with nitrogen gas. The mixture was stirred at r.t. for 10 min. To the flask was added 3 M aq. K_3_PO_4_ 0.400 mL, flushed with nitrogen gas and then the mixture was stirred at 60 °C for 24 h. The resin was filtered and washed with DCM, THF, DMF and MeOH (×3) and finally dried under vacuum yielded 156 mg of resin **6**.

#### 2.3.7. Synthesis of Methyl 1-[4-[5-[4-(N,N-dihexhylamino)phenyl]-2-thienyl]-phenyl]-carboxylate (7)

The synthesized compound on resin was cleaved from 28.9 mg of resin **6**, NaOMe (4.00 mg, 74.0 μmol) with THF 2.40 mL and MeOH 0.600 mL mixture were added to a 15 mL of flask, and flushed with nitrogen gas. The mixture was stirred at reflux for overnight. The resin was filtered and washed with DCM, THF and MeOH. Then the filtrate was dried and purified by silica column chromatography (10% ethyl acetate in hexane) to give compound **7** as a yellow solid (1.60 mg, 21.3%). ^1^H NMR (400 MHz, CDCl_3_) δ (ppm) 8.02 (d, 2H, *J* = 8.6 Hz), 7.66 (d, 2H, *J* = 8.5 Hz), 7.48 (d, 2H, *J* = 8.8 Hz), 7.36 (d, 1H, *J* = 3.9 Hz), 7.14 (d, 1H, *J* = 3.7 Hz), 6.64 (d, 2H, *J* = 8.8 Hz), 3.93 (s, 3H), 3.30 (t, 4H, *J* = 7.6 Hz), 1.57 (m, 4H), 1.33 (m, 12H), 0.91 (t, 6H, *J* = 6.8 Hz); ESI–HRMS (m/z) [M+H]^+^ calcd for C_30_H_40_O_2_NS: 478.27743; found: 478.27721.

## 3. Results

### 3.1. Synthesis of Resin 6 and Dye 7

**Scheme 1:**
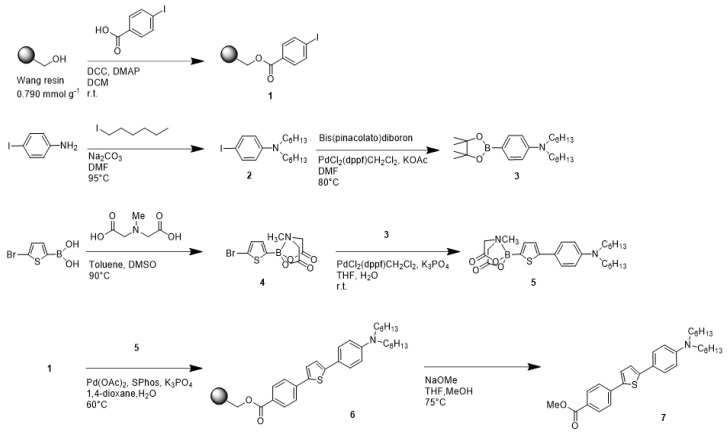
Synthetic schemes for resin **6** and dye **7**.

Resin **6** and dye **7** were synthesized as shown in the synthetic procedure and identified by ^1^H NMR spectra (Scheme 1, Figures S1–S5). The Wang resin was modified with 4-iodobenzoic acid to obtain resin **1**, which was then used as an electron-withdrawing moiety to generate resin **6** by reaction with 5-[4-(*N*,*N*-dihexylamino)phenyl]-2-thienylboronic acid *N*-MIDA ester **5** (electron-donating functionalized hetero aromatic moiety) via Suzuki–Miyaura cross-coupling. For identification and comparison of the product, dye **7** was obtained via transesterification using NaOMe.

**Figure 2:**
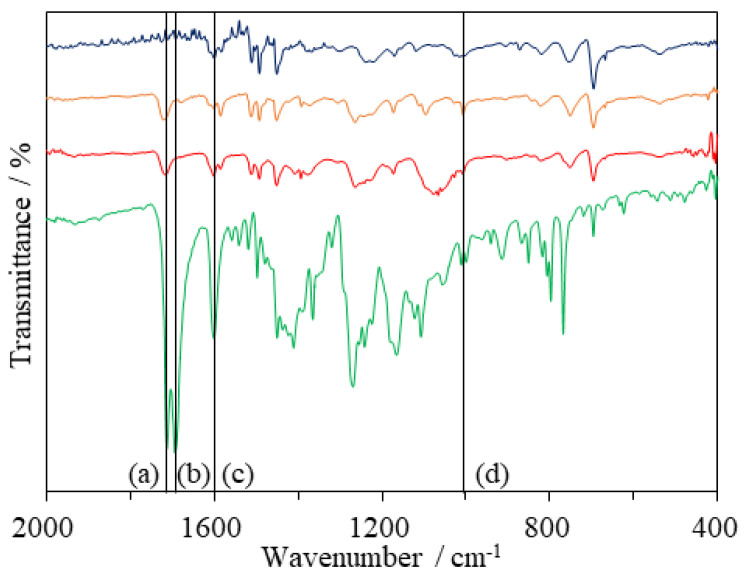
ATR-IR spectra of Wang resin (blue), resin **1** (orange), resin **6** (red), and dye **7** (green) in the dry state. Line (**a**) 1714 cm^−1^; (**b**) 1694 cm^−1^; (**c**) 1600 cm^−1^; (**d**) 1006 cm^−1^.

To confirm the condensation of 4-iodobenzoic acid and the coupling reaction with intermediate **5** on the resin, the ATR-IR spectra of resins **1** and **6**, as well as that of dye **7**, are compared in Figure 1. The peaks at 1714 cm^−1^ from the ester group suggest the condensation of 4-iodobenzoic acid on the naked Wang resin (Figure 2a). In addition, cleaved dye **7** shows several specific peaks from the stretching vibrations in the aromatic ring (Figure 2b–d).

### 3.2. Photophysical Properties of Resin 6 and Dye 7

**Figure 3:**
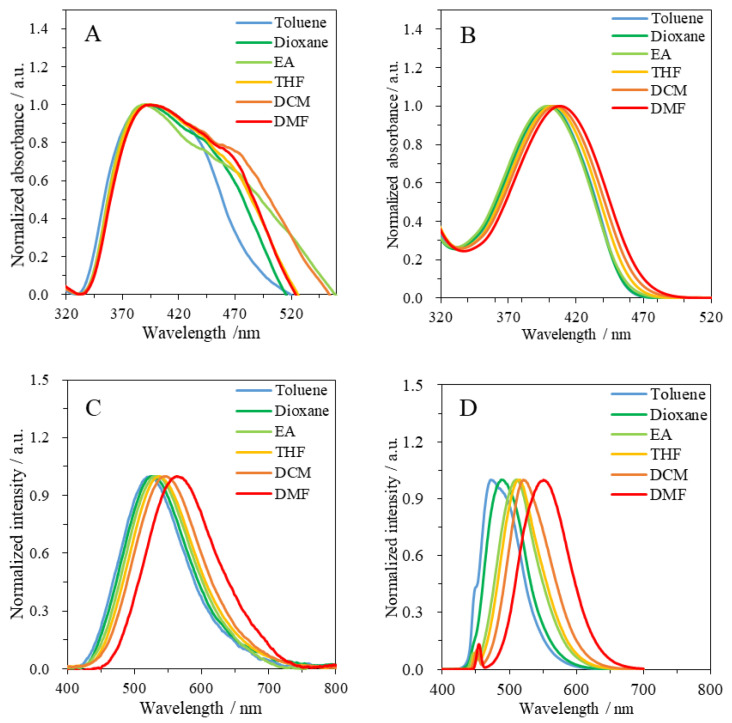
The absorption spectra of resin **6** (**A**), dye **7** (**B**), the emission spectra of resin **6** (**C**), and dye **7** (**D**) in various solvents.

As shown in Table 1, both resin **6** and dye **7** solutions show the absorption maxima around 400 nm in every solvent. Additionally, compared to dye **7**, the absorption spectra of resin **6** rise shoulder peaks above 400 nm. The emission spectra of resin **6** indicate that a spectral shift occurred upon swelling in solvents with different polarities. This spectral shift was in the range of 525–565 nm, whereas that of the dye **7** solution was within 473–551 nm (Table 1, Figure 3). Compared to dye **7** solution, the emission maxima (λ_em_) of resin **6** red-shifted in every solvent (Table 1). The Stokes shift of resin **6** becomes a large value from dye **7** solution (Table 1). Furthermore, the fluorescence quantum yield of resin **6** was lower than those of the dye **7** solutions. These results indicate that resin **6** shows fluorescent solvatochromism, which remains even on the resin surface and the emission bands were red-shifted from the dye **7** solutions in every solvent.

### 3.3. Influence of the Concentration of Immobilized Dye Molecules on the Photophysical Properties

**Figure 4:**
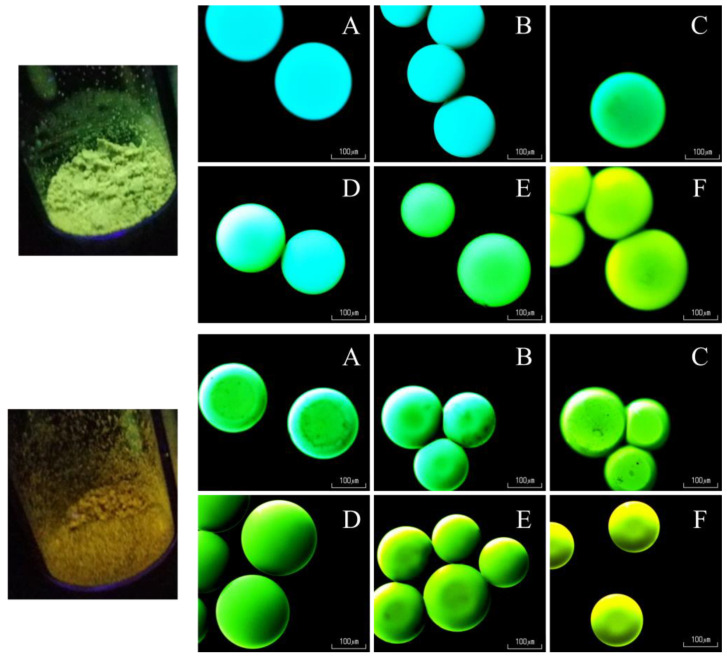
Fluorescence images of resin **6** (above: 0.222 mmol g^−1^, below: 0.545 mmol g^−1^) at the dry state (**left**) and with various solvents (**right**). (**A**) toluene; (**B**) 1,4-dioxane; (**C**) ethyl acetate (EA); (**D**) THF; (**E**) DCM; (**F**) DMF.

**Figure 5:**
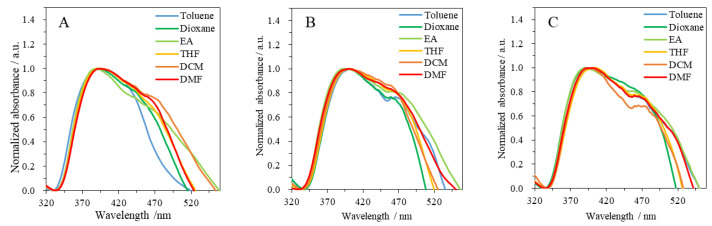
Absorption spectra of resin **6** ((**A**) 0.222 mmol g^−1^, (**B**) 0.342 mmol g^−1^, (**C**) 0.545 mmol g^−1^).

**Figure 6:**
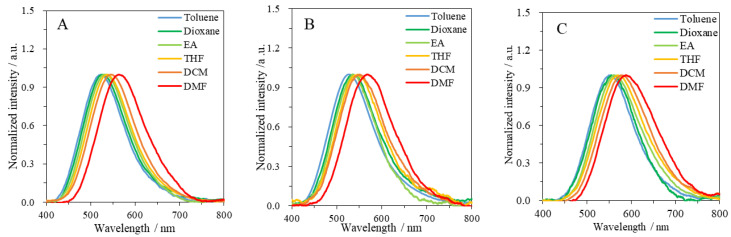
The emission spectra of resin **6** ((**A**) 0.222 mmol g^−1^, (**B**) 0.342 mmol g^−1^, (**C**) 0.545 mmol g^−1^).

To investigate the influence of the dye concentration on its photophysical properties on the solid-phase, the Wang resin was immobilized with various amounts of 4-iodobenzoic acid and then the same procedure was followed as that used to synthesize resin **6**. Different amounts of 4-iodobenzoic acid immobilized resins were prepared as follows: 0.222 mmol g^−1^ were prepared from 5.5 mg 4-iodobenzoic acid and 0.1 g Wang resin. In addition, 0.342 mmol g^−1^ were prepared from 8.5 mg 4-iodobenzoic acid and 0.1 g Wang resin. Furthermore, 0.545 mmol g^−1^ were prepared following the same synthetic procedure as that described for resin **1**; the prepared resins were then used to obtain resin **6** in the same way. The emission color of resin **6** was red-shifted from 0.222 mmol g^−1^ to 0.545 mmol g^−1^ gradually in a dry state (Figure 4, left-side images). Furthermore, resin **6** shows different solvatochromism behavior in various solvents (Figure 4, right-side images). The absorption spectra of resin **6** also show shoulder peaks above 400 nm and emission spectra were red-shifted with increasing concentration of dye molecule on the resin (Figure 5 and Figure 6, Table S1). These results indicated that photophysical properties of resin **6** were influenced by a modified amount of dye molecules.

### 3.4. Emission Spectral Shift for the Co-Solvent

**Figure 7:**
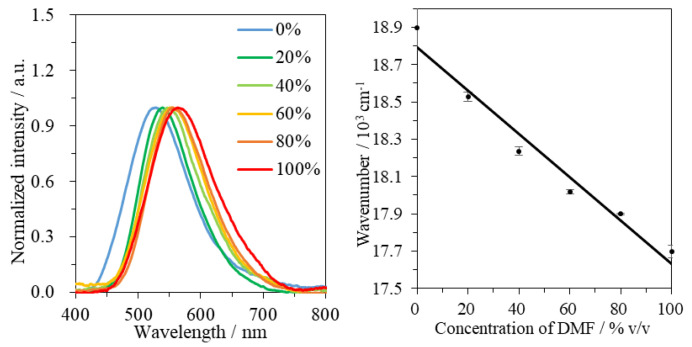
The fluorescence spectra (**left**) and correlation of the emission maxima (**right**) of resin **6** in 1,4-dioxane with different concentrations of the DMF solvent mixture (Concentration of DMF: 0, 20, 40, 60, 80, and 100%, r^2^ = 0.9671).

To demonstrate the ratiometric fluorescence response of resin **6** in a different polarity solvent mixture, we measured its fluorescence spectra in various ratios of 1,4-dioxane and a DMF solvent mixture. With increasing the concentration of DMF, the emission spectrum was shifted to a longer wavelength region (Figure 7, left). The spectral shift almost shows a linear correlation, which indicates that resin **6** exhibits good solvation properties and ratiometric fluorescence response to a solvent mixture (Figure 7, right). As a result, this solid-phase synthesis-and-measurement system is an efficient way for the analytical method to measure the solvent polarity on resin surface. Thus, our fluorescent solvatochromic resin would be applicable to tracing bio-affinity interaction that demonstrates polarity changes on resin surface, such as antigen–antibody interaction [29]. Furthermore, our synthetic strategy would be applied to ratiometric ion sensor material via exchanging the electron-donating moiety using Suzuki–Miyaura cross-coupling based on previous reported fluoroionophores [30].

## 4. Conclusions

In conclusion, fluorescent solvatochromic resin **6** was successfully synthesized by solid surface synthesis, and identified by its photophysical properties, NMR, and ATR-IR spectra. Resin **6** exhibited strong solvatochromic characteristics for the various polarity solvent on the surface, and a ratiometric fluorescence response for the 1,4-dioxane-DMF co-solvent. Following the synthetic strategy, it would be possible to prepare fluorescent sensors for various applications based on the polarity changes on the resin surface. We believe that the synthetic method would facilitate the fabrication of these fluorescent sensor materials, and the combination of the optical waveguide spectrometry and fluorescent sensor materials is a useful method for creating various fluorescent sensor devices.

## Figures and Tables

**Table 1:** Photophysical properties of resin **6** and dye **7** in various solvents.

Solvent	E_T_(30) ^1^/kcal mol^−1^	6				7				
		λ_abs_^2^/nm	λ_em_^3^/nm	Stokes shift/10^3^ cm^−1^	*Φ*_fl_^4^	λ_abs_^2^/nm	λ_em_^3^/nm	Stokes shift/10^3^ cm^−1^	*Φ*_fl_^4^	*ε*^5^/10^4^ M^−1^ cm^−1^
Toluene	33.9	401	525	5.88	0.22	401	473	3.80	0.97	4.19
1,4-Dioxane	36.0	398	529	6.21	0.30	400	489	4.53	0.83	4.47
THF	37.4	401	533	6.20	0.38	403	516	5.42	0.97	4.19
EA ^6^	38.1	394	532	6.61	0.28	399	511	5.47	0.88	4.48
DCM ^7^	40.7	404	548	6.52	0.37	405	521	5.50	1.00	4.14
DMF ^8^	43.2	403	565	7.12	0.36	408	551	6.36	0.91	4.20

^1^ The solvent polarity parameter [28]. ^2^ λ_abs_ represents the absorption maximum. ^3^ λ_em_ represents the emission maximum. ^4^ *Φ*_fl_ represents the fluorescent quantum yield. ^5^ ε represents the molar extinction coefficient. ^6^ EA represents ethyl acetate. ^7^ DCM represents the dichloromethane. ^8^ DMF represents the *N,N*-dimethylformamide.

## Supplementary Materials

The following are available online at https://www.mdpi.com/1996-1944/13/20/4483/s1, Figure S1: ^1^H NMR of compound **2** measured in CDCl_3_ at 400 MHz, Figure S2: ^1^H NMR of compound **3** measured in CDCl_3_ at 400 MHz, Figure S3: ^1^H NMR of compound **4** measured in acetone-d_6_ at 400 MHz, Figure S4: ^1^H NMR of compound **5** measured in acetone-d_6_ at 400 MHz, Figure S5: ^1^H NMR of compound **7** measured in CDCl_3_ at 400 MHz, Table S1: Photophysical properties of resin **6** (0.222 mmol g^−1^, 0.342 mmol g^−1^, 0.545 mmol g^−1^) in various solvents.
