# Supplementary material for: Synthesis of a Fluorescent Solvatochromic Resin Using Suzuki–Miyaura Cross-Coupling and Its Optical Waveguide Spectra to Measure the Solvent Polarity on the Surface
Source: Materials (Basel). 2020 Oct 10;13(20):4483. doi: 10.3390/ma13204483 (PMC7599713; doi:10.3390/ma13204483)
Supplement: Supplementary file 1 [file materials-13-04483-s001.pdf]

materials

MDPI

Supplementary

Synthesis of a Fluorescent Solvatochromic Resin Using Suzuki–Miyaura Cross-Coupling and Its Optical Waveguide Spectra to Measure the Solvent Polarity on the Surface

Yu Otsuka [1], Guanglei Li [2], Hiromi Takahashi [3], Hisashi Satoh [4], and Koji Yamada [1,5,*]

[1] Division of Materials Science, Graduate School of Environmental Science, Hokkaido University, Sapporo, Hokkaido 060-0810, Japan; y_outsuka214@eis.hokudai.ac.jp

[2] School of Food Science and Technology, Nanjing University of Finance & Economic, Nanjing 210023, China; liglhit@gmail.com

[3] ATR Scientists Partners Inc., 3-27-13, Maehara, Koganei, Tokyo 184-0013, Japan; atr.scientists.partners.inc.1995@gmail.com

[4] Division of Environmental Engineering, Faculty of Engineering, Hokkaido University, Sapporo, Hokkaido 060-8628, Japan; qsatoh@eng.hokudai.ac.jp

[5] Division of Materials Science, Faculty of Environmental Earth Science, Hokkaido University, Sapporo, Hokkaido 060-0810, Japan

* Correspondence: yamada@ees.hokudai.ac.jp; Tel.: +81-011-706-2254

Received: 18 September 2020; Accepted: 2 October 2020; Published: 10 October 2020

1. ^{1}H NMR spectra of compound 2-5, and 7

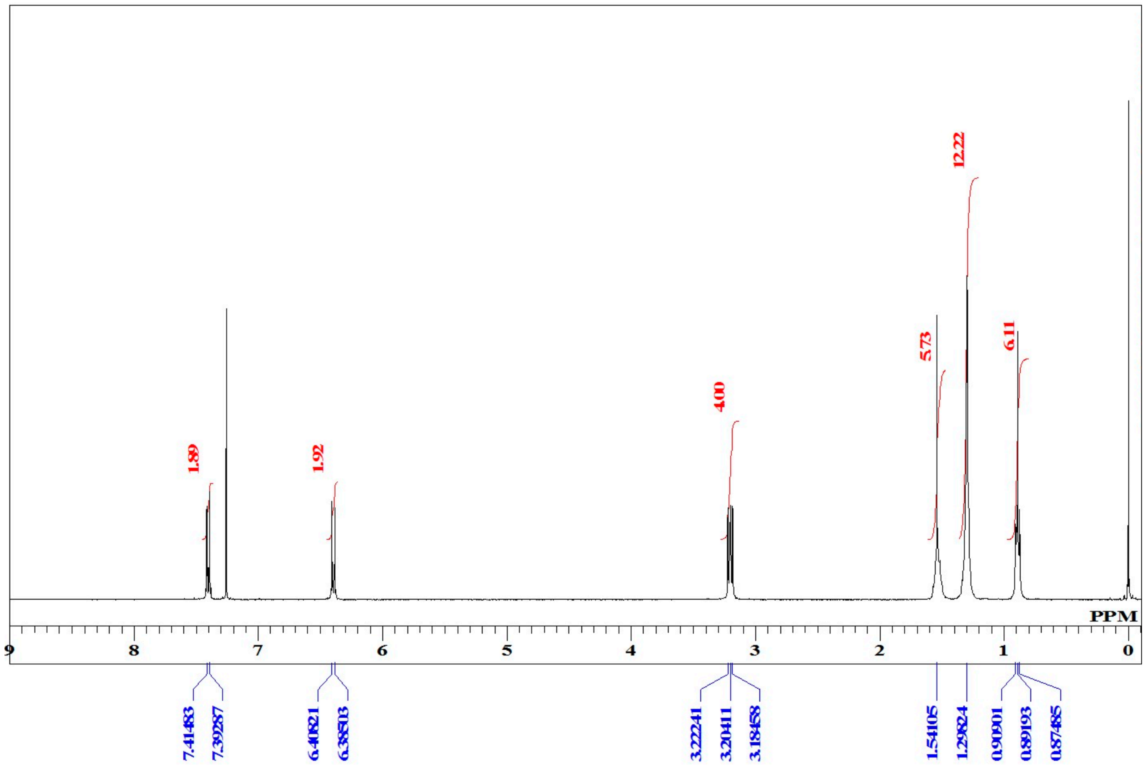

Figure S1. ^{1}H NMR of compound **2** measured in $CDCl_3$ at 400 MHz.

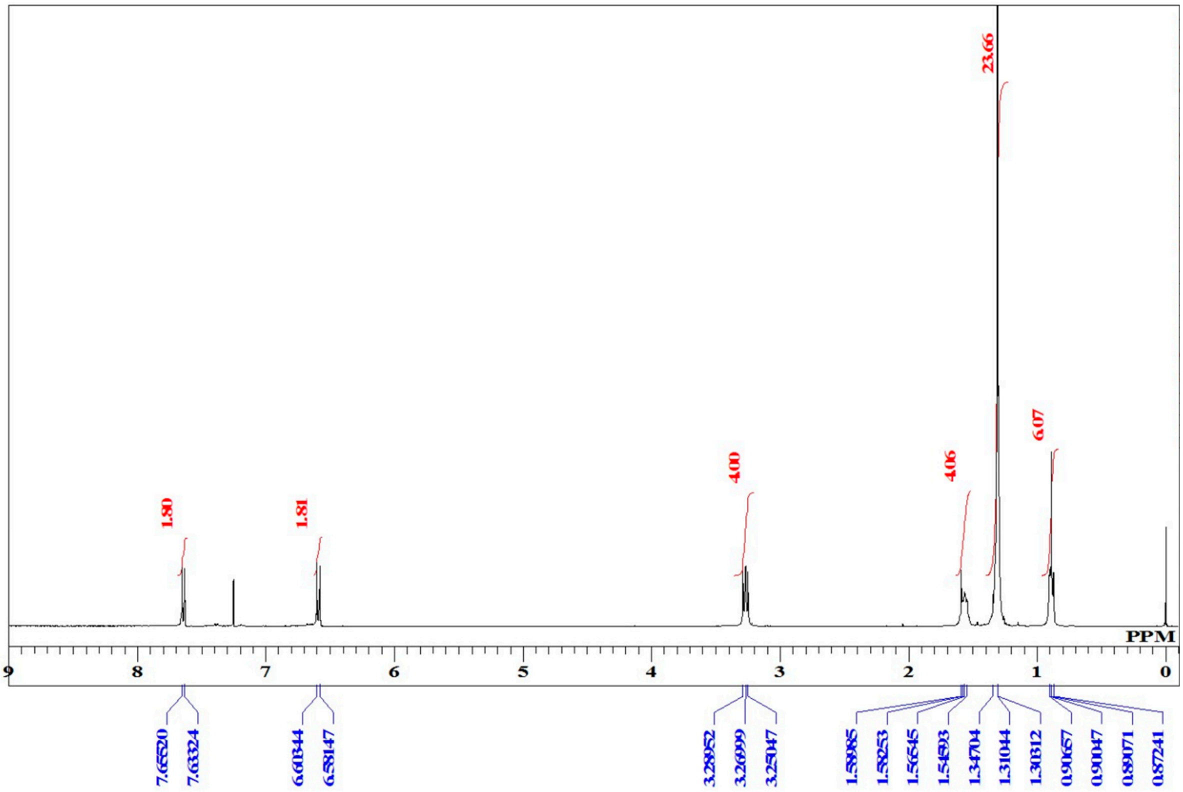

Figure S2. ^{1}H NMR of compound **3** measured in $CDCl_3$ at 400 MHz.

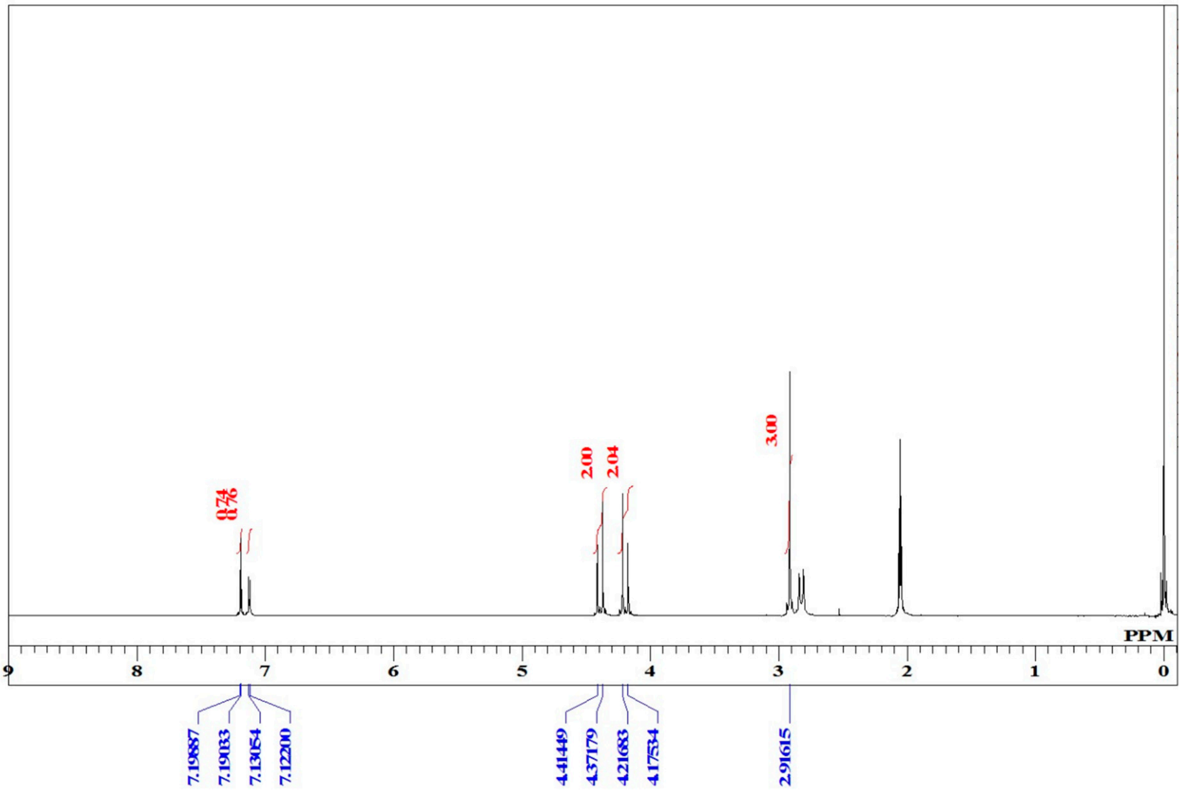

Figure S3. ^{1}H NMR of compound **4** measured in acetone-d_6 at 400 MHz.

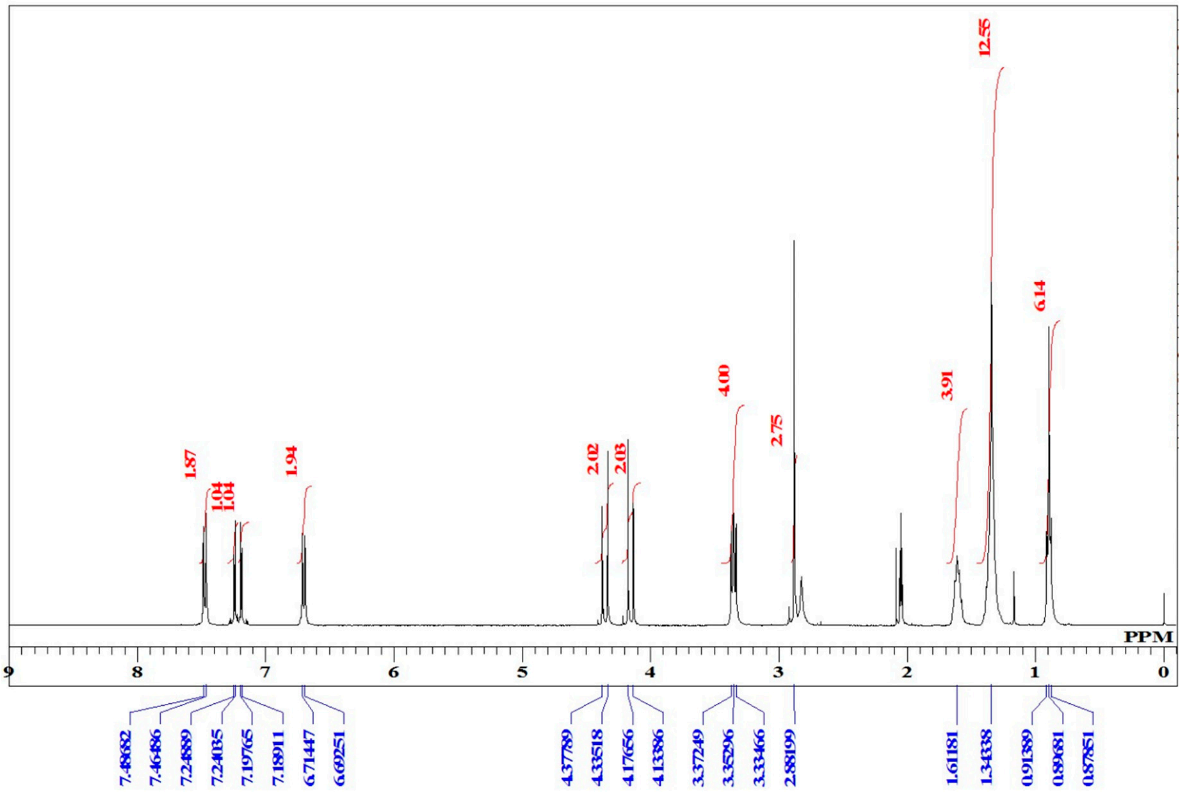

Figure S4. ^{1}H NMR of compound **5** measured in acetone-d_6 at 400 MHz.

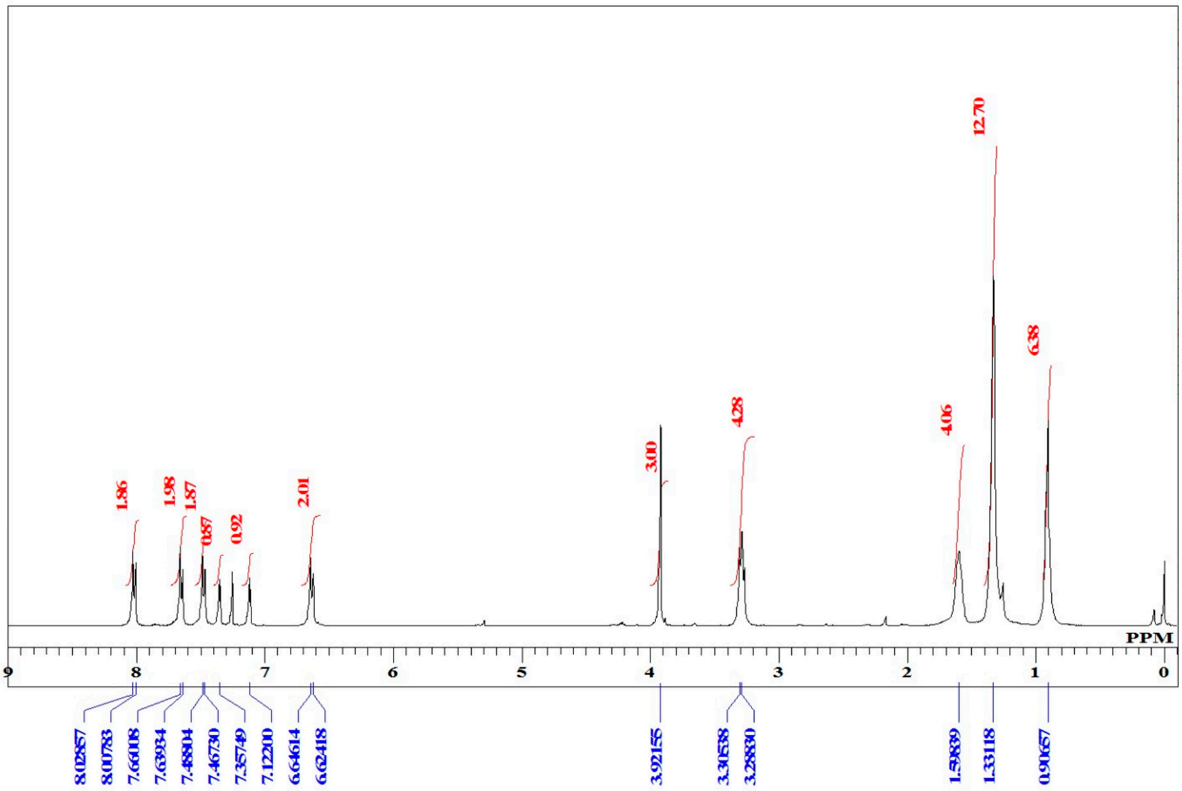

Figure S5. ^{1}H NMR of compound **7** measured in $CDCl_3$ at 400 MHz.

2. Photophysical properties of resin 6

Table S1. Photophysical properties of resin **6** (0.222 mmol g^{-1}, 0.342 mmol g^{-1}, 0.545 mmol g^{-1}) in various solvents.

**Solvent**	**$E_T(30)^a$ / kcal mol^{-1}**	**0.222 mmol g^{-1}**			**0.342 mmol g^{-1}**			**0.545 mmol g^{-1}**		
		**λ_{abs}^b / nm**	**λ_{em}^c / nm**	**Stokes shift / 10^3 cm^{-1}**	**λ_{abs}^b / nm**	**λ_{em}^c / nm**	**Stokes shift / 10^3 cm^{-1}**	**λ_{abs}^b / nm**	**λ_{em}^c / nm**	**Stokes shift / 10^3 cm^{-1}**
Toluene	33.9	401	525	5.88	404	530	5.87	404	548	6.51
1,4-Dioxane	36.0	398	529	6.21	403	531	5.99	399	550	6.88
THF	37.4	401	533	6.20	410	546	6.10	405	559	6.82
EA[d]	38.1	394	532	6.61	404	544	6.38	401	561	7.13
DCM[e]	40.7	404	548	6.52	407	552	6.48	399	568	7.44
DMF[f]	43.2	403	565	7.12	401	566	7.26	403	583	7.69

[a] The solvent polarity parameter [1]. [b] λ_{abs} represents the absorption maximum. [c] λ_{em} represents the emission maximum. [d] EA represents ethyl acetate. [e] DCM represents the dichloromethane. [f] DMF represents the *N,N*-dimethylformamide.

References

1. Reichardt, C. Solvatochromic dyes as solvent polarity indicators. *Chem. Rev.* **1994**, 94, 2319-2358, doi: 10.1021/cr00032a005.

© 2020 by the authors. Licensee MDPI, Basel, Switzerland. This article is an open access article distributed under the terms and conditions of the Creative Commons Attribution (CC BY) license (http://creativecommons.org/licenses/by/4.0/).
